# Cooccurrence of Metastatic Papillary Thyroid Carcinoma and* Salmonella* Induced Neck Abscess in a Cervical Lymph Node

**DOI:** 10.1155/2017/5670429

**Published:** 2017-02-05

**Authors:** Jae-Myung Kim, Ju-Yeon Kim, Eun Jung Jung, Eun Jin Song, Dong Chul Kim, Chi-Young Jeong, Young-Tae Ju, Young-Joon Lee, Soon-Chan Hong, Sang-Kyung Choi, Woo-Song Ha

**Affiliations:** ^1^Department of Surgery, Gyeongsang National University School of Medicine and Gyeongsang National University Hospital, Jinju, Republic of Korea; ^2^Department of Surgery, Gyeongsang National University School of Medicine and Gyeongsang National University Changwon Hospital, Changwon, Republic of Korea; ^3^Department of Pathology, Gyeongsang National University School of Medicine, Gyeongsang Institute of Health Sciences, Gyeongsang National University, Jinju, Republic of Korea

## Abstract

Cervical lymph node metastasis is common in patients with papillary thyroid carcinoma (PTC).* Salmonella* species are rarely reported as causative agents in focal infections of the head and neck. The cooccurrence of lymph node metastasis from PTC and a bacterial infection is rare. This report describes a 76-year-old woman with a cervical lymph node metastasis from PTC and* Salmonella* infection of the same lymph node. The patient presented with painful swelling in her left lateral neck region for 15 days, and neck ultrasonography and computed tomography showed a cystic mass along left levels II–IV. The cystic mass was suspected of being a metastatic lymph node; modified radical neck dissection was performed. Histopathological examination confirmed the presence of PTC in the resected node and laboratory examination of the combined abscess cavity confirmed the presence of* Salmonella Typhi*. Following antibiotic sensitivity testing of the cultured* Salmonella Typhi*, she was treated with proper antibiotics. Cystic lesions in lymph nodes with metastatic cancer may indicate the presence of cooccurring bacterial infection. Thus, culturing of specimen can be option to make accurate diagnosis and to provide proper postoperative management.

## 1. Introduction

PTC is the most common well-differentiated cancer of the thyroid gland, accounting for 80–85% of well-differentiated thyroid malignancies [1]. PTCs generally show a good prognosis, although they metastasize to regional lymph nodes in 30–80% of patients [2].

The simultaneous presence of infection and metastasis in the same lymph node is rare, with most cases observed in patients with tuberculosis [3–8]. This report describes a patient with a PTC-derived metastasis coexisting with an abscess due to* Salmonella* infection.

## 2. Case Presentation

A 76-year-old female presented with painful swelling in the left lateral neck region for 15 days. There was no erythema or heating sensation on neck area, and she had no symptoms of fever. Ten months earlier, she had undergone total thyroidectomy with central neck lymph node dissection due to PTC (T3N1aM0 by American Joint Committee on Cancer/International Union against Cancer Staging System 7th Edition), followed by radioactive iodine therapy and maintenance treatment with levothyroxine.

Thyroid function tests showed mildly suppressed thyrotropin-stimulating hormone (TSH; 0.01 mIU/L; normal range, 0.27–4.2 mIU/L) and elevated free thyroxine (fT4; 2.12 ng/dL; normal range, 0.93–1.70 ng/dL) concentrations. Her white blood cell and neutrophil counts were normal. No pathogens were cultured from her blood, stool, urine, or sputum.

**Figure 1:**
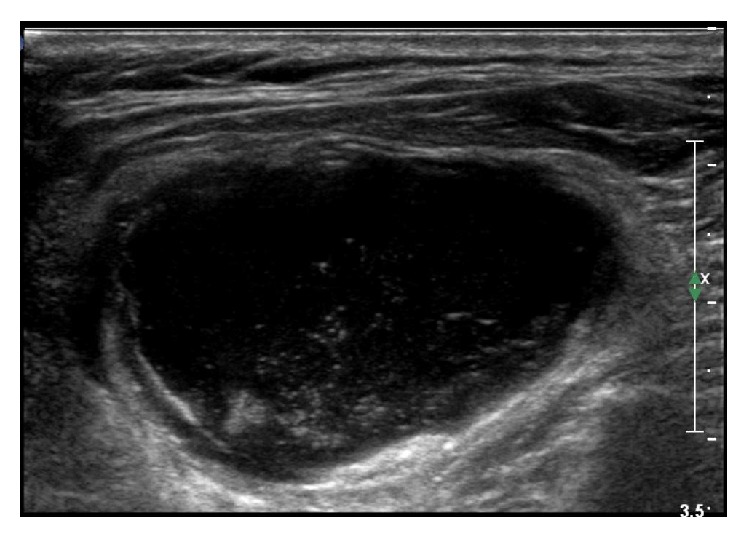
Ultrasonography showing a large heterogeneously echoic mass.

**Figure 2:**
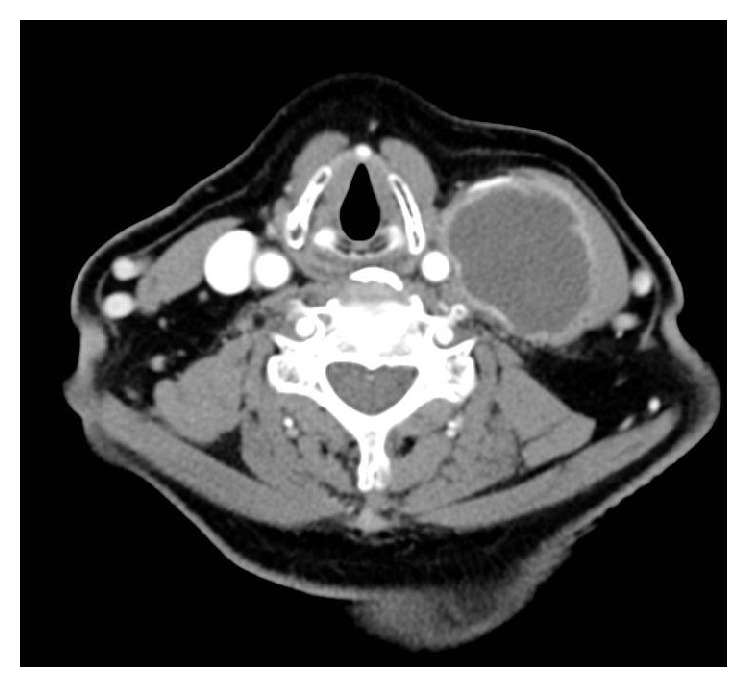
Computed tomography showing a 3.3 × 5.6 cm sized thick-walled cystic lesion.

**Figure 3:**
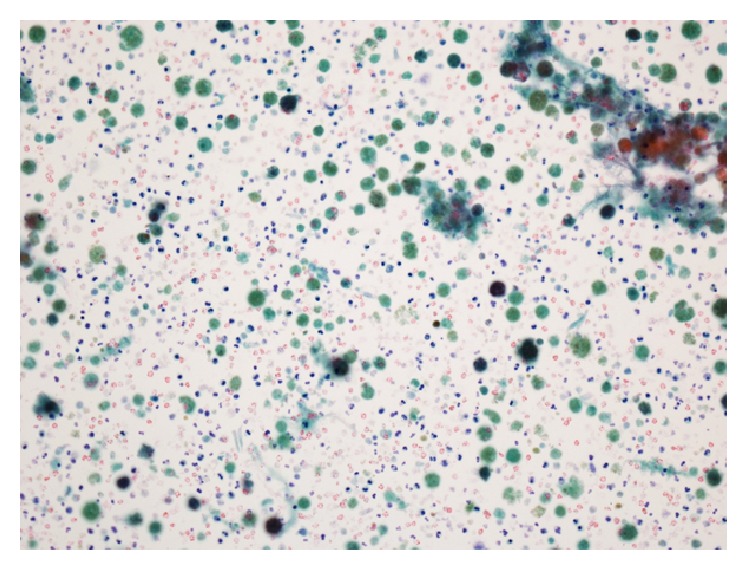
Cytologic features of the collected fluid by fine-needle aspiration (×200).

Neck ultrasonography revealed a large heterogeneous echoic (cystic) mass in the left lateral neck (Figure 1) and computed tomography revealed a 3.3 × 5.6 cm sized thick-walled cystic lesion along left levels II, III, and IV (Figure 2). Cytologic examination of the dark brown colored fluid collected from the lesion by fine-needle aspiration showed many neutrophils and macrophages in necrotic background, but there was no evidence of malignancy (Figure 3). However, the mass was suspected of being a metastatic lymph node and was resected.

**Figure 4:**
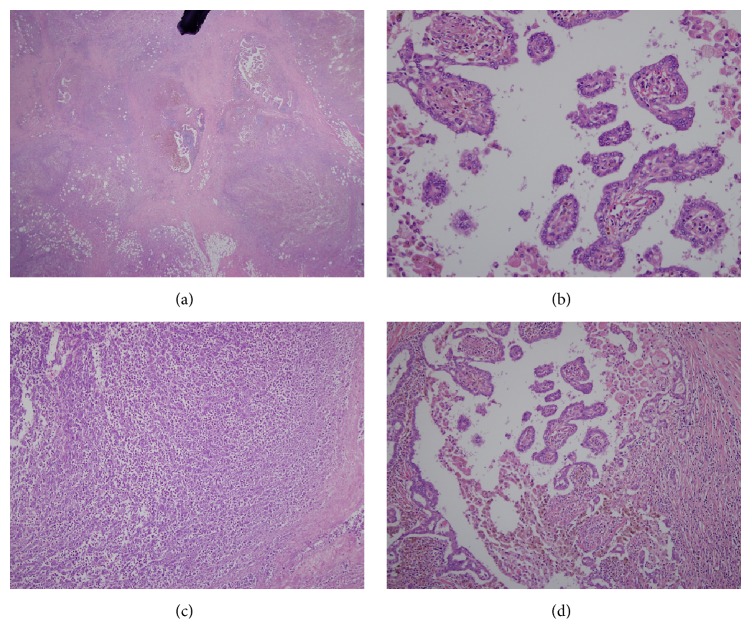
(a) Panoramic view of the resected node which contains the papillary carcinoma and necrotic debris (H&E stain, ×25). (b) Histological characteristics of the papillary cancer (H&E stain, ×200). (c) Histological characteristics of the abscess (H&E stain, ×100). (d) Histological characteristics of the papillary cancer (H&E stain, ×100).

Intraoperative examination showed that mass was encapsulated and very highly adhesive to the internal jugular vein and around the tissue. There were no other abnormally enlarged lymph nodes. The mass contained dark brown colored fluid and some debris. Microscopic examination of the lymph node mass showed massive necrosis and immunohistochemistry clearly showed that the mass was a metastatic papillary carcinoma (Figure 4). Culture of the fluid showed the presence of* Salmonella Typhi*.

No postoperative complications occurred. The patient was treated with intravenous cephalosporin for 6 days; following antibiotic sensitivity testing of the cultured* Salmonella Typhi*, she was switched to oral ciprofloxacin for 7 days. Regular follow-up examinations have shown no evidence of additional metastasis, recurrence of metastasis, or residual infection.

## 3. Discussion

Nodal metastasis of PTC can appear as a solid or cystic mass. Approximately 40% of lymph node metastases from PTCs tend to cavitate via complete cystic degeneration [9]. This cystic lymph node metastasis may therefore have been misinterpreted as a benign cervical cystic mass.

*Salmonella* is a nonencapsulated Gram negative motile bacillus.* Salmonella* infection may lead to typhoid fever, enterocolitis, or bacteremia with focal lesions in various parts of the body [10, 11].

Head and neck infection normally arise from* Streptococcus*,* Staphylococcus*,* Haemophilus*, or other anaerobic species.* Salmonella* infections of the head and neck are usually found in the tonsils, middle ear, and parotid gland but are rarely reported in neck abscess [10–13]. In one case report,* Salmonella Typhi* was isolated from the blood of a 20-year-old Korean woman with persistent fever, headache, myalgia and cervical lymphadenopathy, with this patient finally diagnosed with necrotizing lymphadenitis due to* Salmonella Typhi* [14].

Conventional treatment of a* Salmonella* neck abscess includes incision and drainage, along with treatment with appropriate antibiotics [15]. Recently, quinolones and third-generation cephalosporins were shown to be effective as first-line treatment against* Salmonella* [16].

Lymphadenitis has been reported to be an extraintestinal manifestation of* Salmonella* infection in patients with malignancy.* Salmonella* was isolated from a supraclavicular node in a patient with stomach cancer and from an axillary lymph node in a patient with reticulum cell sarcoma [17].* Salmonella Braenderup* was isolated from a cervical lymph node of a patient with Hodgkin's lymphoma [18], and group D nontyphoidal* Salmonella* was isolated from a cervical lymph node, positive for diffuse large B cell lymphoma [19]. In the present case,* Salmonella Typhi* was isolated from the same lesion containing a metastatic PTC; to our knowledge, this is first case report describing the isolation of* Salmonella* from a metastatic lymph node associated with PTC.

In summary, a* Salmonella* species was able to infect a cervical lymph node and progress to neck abscesses in patients with PTC, and cervical lymph node metastasis is common in patients with PTC. The presence of an abnormally enlarged neck mass in a patient with PTC may suggest the cooccurrence of* Salmonella* infection and malignancy in the same lesion. Culturing of the specimen may enhance diagnostic accuracy and providing proper postoperative management.
